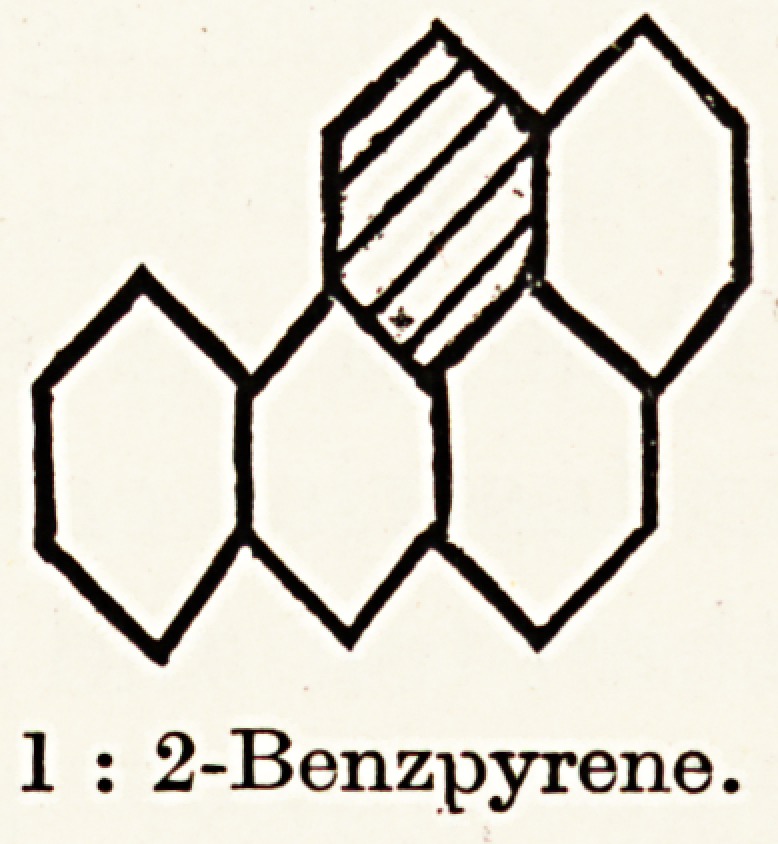# The Twenty-Second Long Fox Memorial Lecture: Recent Advances in the Ætiology, Diagnosis and Treatment of Cancer

**Published:** 1933

**Authors:** Cecil A. Joll

**Affiliations:** Senior Surgeon to the Royal Free Hospital; Surgeon to the Cancer Hospital


### The Bristol Medico-Chirurgical Journal

*"Scire est nescire, nisi id me
Scire alius sciret."*

WINTER, 1933.

### THE TWENTY-SECOND LONG FOX MEMORIAL LECTURE:

DELIVERED IN THE UNIVERSITY OF BRISTOL ON NOVEMBER 7TH, 1933.

THE VICE-CHANCELLOR (Dr. T. LOVEDAY, M.A., LL.D.) *in the Chair.*

BY

CECIL A. JOLL, M.D., M.S., F.R.C.S.,

*Senior Surgeon to the Royal Free Hospital; Surgeon to the Cancer Hospital,*

ON

### RECENT ADVANCES IN THE ÆTIOLOGY, DIAGNOSIS AND TREATMENT OF CANCER.

THE Bristol Medical School has always had its great teachers, so that something more than the capacity to instruct must account for the outstanding position which Long Fox held in the medical world for over a quarter of a century. A fine character, combined with a striking personality, formed the basis of a

R

JAN 2 3 1934

reputation which had made his name famous in the West Country when I was a schoolboy, and has so endured that to-day we meet to do honour once again to his memory.

### ÆTIOLOGY.

Broadly, it may be said that there are two main hypotheses as to the real nature of cancer and the cancerous process: one, that it is a single disease comparable, for example, to syphilis; the other, that it is a complex of diseases, some as obviously closely related to one another as are the various species of a genus in some homogeneous natural order of plants or animals, others as obscurely connected as are certain natural orders themselves.

It follows that those who hold the former view expect to be able to find a single causative factor for cancer, and that in time a universal diagnostic method will be forthcoming which will reveal each and every malignant growth at its earliest stage. The logical outcome, it is assumed, would then follow, that is, a cure equally lethal for all types of neoplasm. Those who accept the latter hypothesis are prepared to find that progress in our knowledge of cancer is made bit by bit, rather than by any dramatic achievement which, at a bound, would justify our hopes of bringing malignant neoplasia into the category of a controllable disease.

Twenty years spent in intimate association with the natural history of the cancer problem, particularly in its clinical and pathological aspects, has converted me to the second of the alternative hypotheses, and thus I am compelled to be critical—I hope not hyper-critical—towards the claims to diagnose cancer by a single test or to cure it by a specific agent.

In the endeavour to appraise progress in our knowledge of the causation of cancer one is at once impressed by the tendency to confuse the exciting cause — the "causa causans" — with the disease phenomena.

Thus it appears to me a fundamental error to attribute cancer, as did Farmer, Moore and Walker,[1] to sex-cell mitosis or to this or that peculiarity of the chromosome or nuclear material; one and all—even if it could be proved that they occur in every malignant growth — they are merely the cytological phenomena of the disease, and not its cause. A similar criticism must apply to Sampson Handley's[2] thesis that chronic lymph-stasis is the fundamental factor in carcinogenesis. He maintains that all carcinomata are preceded by papillomata, which are themselves the outcome of lymph stasis induced by chronic inflammation in the papillary lymphatics. No evidence, however, is brought forward of a similar cause for sarcomata, yet, as I shall show later, both carcinoma and sarcoma can be induced by the same carcinogenic compound. It is, moreover, extremely unlikely that there is any radical difference in the mode of action of such compounds on epithelial and connective tissues respectively. Handley's carefully considered and lucid thesis must be recognized as throwing a valuable side-light on the histology of the cancer process, but in no real sense clarifying the vexed question of its causation.

The skill of the bacteriologist and the pathologist have been fully tested by those who believe that cancer is comparable with a micro-organismal or parasitic disease, but I need not remind you that not one of the numerous theories in this group has withstood destructive criticism. It is, of course, true that Gye and Purdy[3] have made out a strong case for the belief that the Rous sarcoma of fowls is transmitted by a filtrable virus, the growths appearing extremely rapidly after inoculation. Nothing comparable with this is found in any mammalian cancer, and up to now Rous's sarcoma is to be classed merely as an interesting anomaly among neoplasms.

It is well established that certain metazoan parasites are capable of behaving as carcinogenic agents. Well-authenticated examples of this association are carcinoma of the stomach in rats, which Fibiger[4] so brilliantly established to be due to a nematode (Spiroptera) carried to the stomach wall by a particular cockroach, and a sarcomatous tumour of the liver in rabbits produced by a cysticercus and carcinoma of the equine testis caused by a nematode. It must also be remembered that malignant disease at certain sites, and in particular animals, may in fact be due to a unicellular protozoan, the best known being the adeno-carcinoma of the liver of the rabbit in association with coccidiosis. Not one of these parasites should, I think, be regarded in any other light than as a carcinogenic agent comparable to the various physical and chemical agents which I shall shortly proceed to discuss.

Clinicians have for many years maintained that cancer frequently arises at sites liable to what they term "chronic irritation." I give you the term, as lawyers would say, "without prejudice," since there is no unanimity among pathologists as to whether in fact the change preceding the development of cancer at such sites actually is of a chronic inflammatory type. Furthermore, it must not be forgotten that many chemical irritants exist, *e.g.* chromic acid, which produce industrial skin diseases but do not lead to epithelioma. In recent years, too, there have been recorded many cases of cancer ascribed to the action of heat (Kangri burns), sunlight, X-rays, radium,[5] coal tar, paraffin and lubricating oils, aniline dyes,[6] arsenic and betel-nut.

Experimentally, the whole subject of carcinogenetic agents received a great stimulus by the discovery of Yamagiwa and Ichikawa[7] in 1914 that epithelioma can be produced by painting the ear of the rabbit with coal-tar. After the War a considerable series of positive results were obtained, chiefly in mice and rats, by a variety of other agents—radium,[8] X-rays,[9] ultra-violet light,[10] sunlight,*

* International Cancer Congress, Madrid, 1933.

dental tartar,[11] printing ink,[12] $CO_2$ snow,[13] paraffin,[14] folliculin.*

It is to be noted specially that in all cases the interval which elapses between the initiation of the use of the agent and the appearance of the tumour is considerable, relative to the life of the animal concerned. This is in strict accordance with the findings in industrial cancer, in which the interval is seldom less than ten and may be as much as fifty years. Leitch[15] has stated that in experimental tar-carcinogenesis there is the same incubation period whatever the age of the animal, and this is largely borne out by statistical inquiries in occupational cancer, as it occurs in aniline and tar workers, etc. It is of interest also to note that not only does a diet of liver increase the growth in tar cancers (Maisin and François[16]), but it also accelerates their appearance (Watson[17]).

During the last few years a series of brilliant researches aimed at elucidating the problems of tar-carcinogenesis has been brought to fruition at the Research Institute of the Cancer Hospital, and it would be difficult to exaggerate their importance. The full details of this work are too technical for me to attempt to describe, but as an example of the value of team work in cancer research and of the great assistance which the cognate sciences can render to medicine, I feel justified in giving you a brief outline of this remarkable achievement.†

In 1927 Mayneord, while examining the fluorescence spectra of tar and shale oil, observed in them three very definite bands, one rather narrow and two wider near the limit of the visible rays. A systematic spectroscopic survey of various

* Lacassagne, personal communication, 1933.

† I have to thank my colleague, Professor Kennaway,[18] Director of the Research Laboratories, for supplying much of the information relative to this part of my lecture.

hydrocarbons next enabled him to detect a somewhat similar arrangement of bands in the spectrum of 1 : 2 benzanthracene,

1 : 2 benzanthracene.

1 : 2 : 5 : 6 benzanthracene.

1 : 2-Benzpyrene.

but this substance was found to be almost without cancerogenic powers. When, however, 1 : 2 : 5 : 6 benzanthracene was examined spectroscopically the bands were found to approximate still more closely to that of tar and shale-oil, and it proved to be strongly carcinogenic.

It was now realized that there were great possibilities along this line of investigation, and it was therefore attacked on a large scale. Hieger,[19] starting with two tons of tar, eventually obtained 50 grammes of a crystalline substance. This was found by Cook[20] to be 1 : 2 benzpyrene, a hitherto unknown substance. Cook[21] and Hewett[22] succeeded in synthesizing this compound and subsequently found that :—

1. Its spectrum is identical with that of tar and shale-oil.

2. It produces numerous growths, both carcinoma and sarcoma, in rats and mice.

Thus it has been established that an actively carcinogenic compound of known composition 1 : 2 benzpyrene occurs in tar and shale-oil, and is almost certainly the principal, if not the only active carcinogenic agent in them. (The other synthetic hydrocarbon, 1 : 2 : 5 : 6 benzanthracene, has never been *proved* to exist as such in tar and shale-oil.) More recently the same group of workers have synthesized two other actively carcinogenic compounds, viz. 5 : 6 cyclo-penteno—1 : 2 benzanthracene and 3 : 4 benzphenanthrene (chemically this latter is the simplest carcinogenic compound thus far known).

Further work is now being undertaken by Mayneord[23] to determine by spectroscopic methods, if possible, what types of molecular structures are concerned in the capacity of a substance to induce cancer, and valuable information has also been obtained in this direction by the aid of X-ray crystallography in Sir W. Bragg's laboratory. When this is completed it may be possible to prove whether such substances as sterol and bile acids, occurring normally in the human body, could become converted under abnormal metabolic conditions into one or other of the carcinogenic hydrocarbons.

We know, for example, that the rut-producing hormone oestrin (or folliculin) is, chemically, probably an aromatic substance, and further, from the urine of pregnant mares an œstrogenic compound of the aromatic series can be isolated. Recently Lacassagne, of Paris, showed me cancers of the breast produced in male mice by fortnightly injections of folliculin, starting these injections when the mice were a week or two old. It is a remarkable fact, too, that 5 : 6 cyclo-penteno, 1 : 2 benzanthracene and 1 : 2 benzpyrene are not only carcinogenic but also œstrogenic.

Before passing on to discuss the hereditary factor in cancer it is, I think, proper to remind you that the identification of the carcinogenic substance in tar and shale-oil responsible for one type of industrial epithelioma emphasizes the probability of a multiplicity of causes of cancer, and helps to refute the claims of those who would ascribe cancer to a single cause.

*Heredity.*

Whatever may eventually be revealed as to the immediate exciting cause of cancer, nobody can be indifferent to the question: Is it inherited, and if so, under what circumstances? As practical clinicians we cannot fail to be impressed by the significance of heredity in connexion with disease and disease tendencies generally. By analogy, therefore, a strong case can be made out for an hereditary basis in malignant disease. The occurrence of certain tumours in identical twins provides a concrete example; thus H. Gideon Wells[24] records two such sets, all four of whom died of malignant disease of the testicle, the testicle affected and the histological structure of the tumour being identical in each twin. When, however, an attempt is made to formulate the exact or even the approximate nature of the genetic influence in cancer production, we find ourselves at once involved in a problem of such complexity that up to now no single unassailable generalization on the subject has been evolved.

The careful experimental work of Slye,[25] continued as it has been over many generations in mice, led her to conclude that for certain spontaneous growths of the breast the tendency is for the disease to be inherited as a Mendelian recessive.

The unlikelihood of cancer-susceptibility being transmitted as a simple dominant was indicated by Tyzzer[26] as far back as 1907. He pointed out that if cancer were inherited as a single factor (or single gene) dominant, the crossing of an insusceptible individual with one homozygotic for cancer would result in the production of offspring in all of which the tumour character would appear. Such an occurrence, had it in fact ever happened, would certainly have been recognized clinically ere now. Little[27] has criticized Slye's conclusions severely, but without, I think, successfully refuting them *in so far as the particular type and site of the cancer* in Slye's series is concerned.

There is ample ground, however, for rejecting the idea that cancer, generally, is inherited in man as a simple recessive or simple dominant ; but, nevertheless, there are one or two instances in human pathology in which malignant growths do indeed appear to be transmitted as simple gene dominants or simple gene recessives.

| Human Tumours Transmitted as Mendelian Dominants. | Human Tumours Transmitted as Mendelian Recessives. |
|---|---|
| Retinoblastoma.<br>Multiple Neurofibromata.<br>Multiple Adenomata (polyposis of colon). | Malignant transformation of Xeroderma pigmentosum.<br>Subcutaneous Lipomata (sex-linked). |

These and a few others comprise all the tumours which appear to be inherited by simple genes, and it therefore seems to be more reasonable, as Haldane[28] suggests, that the genetical predisposition to malignant disease should be ascribed to multiple dominant genes.

It is, however, I think, fair to state that the investigations of the past twenty years indicate that cancer or, rather, cancer-susceptibility in man is undoubtedly inherited, but that no facts are available at present which make it feasible to minimize this susceptibility by any form of selective breeding such as is included under the term "eugenics."

In concluding this section of my lecture it should be put on record that as our knowledge of the natural history of cancer widens, particularly with regard to the occurrence of spontaneous tumours in wild and domesticated animals and among the less accessible races of mankind, more and more evidence is accumulating that race, religion, diet, moderate indulgence in alcohol or tobacco have no influence whatsoever on the causation of cancer. The refutation thus afforded of the loudly-proclaimed hypotheses of quacks, charlatans and fanatics may perhaps in time aid in checking the spate of fantastic suggestions, which serve only to confuse the issue in the minds of the general public while providing fresh material for exploitation by the sensational press.

### DIAGNOSIS.

Few will quarrel with the statement that success in cancer therapy lies in the earliest possible diagnosis. One of my lay friends has expressed this rather aptly, I think, in the slogan : "Fear not cancer, fear delay." Those of us who are actively engaged in treating malignant disease have opportunities daily to witness the tragedy of procrastination on the part of the patient, and not always the poor and ignorant one; but this is not the whole story. As a profession we are not yet sufficiently tuned up to be constantly suspicious of the existence of cancer. I have tried to avoid thus far the appeal to statistics to illustrate my points, but here it seems to be inevitable. Fortunately, they are relatively simple and unequivocal.

MacCarty[29] reports that from 30 per cent. to 50 per cent. of all cases of cancer of the breast seen at the Mayo Clinic over a period of years were inoperable at their first examination, the figures for the large bowel and stomach being 42 per cent. and 75 per cent. respectively. My own experience fully confirms these statistics, except in connexion with carcinoma of the stomach, in which during the last twelve years less than 20 per cent. have proved operable, the tendency recently being, I regret to say, for the percentage to fall rather than rise, the direct outcome, I believe, of the misguided enthusiasm for the alkaline treatment of all forms of dyspepsia. Only when the lesion has unmistakably declared itself as malignant is the diagnosis of simple gastric ulcer abandoned. MacCarty states further that less than 25 per cent. of the cases of gastric and colic cancer had been X-rayed before reaching the Clinic, and 20 per cent. of the rectal and sigmoid growths had shortly before been treated for such conditions as piles and fissure in ano and the malignant lesion entirely overlooked.

De Vries,[30] of Amsterdam, found that 20 per cent. of cancer cases had not been recognized previous to autopsy, and conversely 10 per cent. diagnosed as cancer were found free from malignant disease. Closely comparable figures are provided by the Imperial Cancer Research Commission[31] in England and by Bilz[32] in Germany. Naturally, the greater percentage of errors occurred in connexion with deep-seated growths, but it must be obvious that as the figures relate to autopsy material, presumably the percentage error at the earlier stages is very much higher.

It is also significant that Reimann and Safford,[33] surveying 3,780 cases of cancer over a period of twenty-eight years, could find little evidence of improvement in the delay in seeking proper advice, even towards the end of the period.

It is impossible to face these facts without concluding that we are not educating our students and practitioners to suspect cancer often enough. It must be borne in mind that the number of cases of malignant disease met with by a general practitioner is relatively small; for if we assume that 75,000 cases occur per annum, and that there are 25,000 doctors in general practice in this country, this gives an annual average of three cases apiece. Unless, therefore, the student of medicine, undergraduate or graduate, receives special instruction in the diagnosis of cancer and is given opportunities for the periodical revision of his knowledge, little progress will be made, however great the resources available in the hands of the trained cancer expert. It is useless to equip a factory with the most modern machinery if the appropriate raw material is lacking. Universities with medical schools and the special cancer hospitals must stimulate and organize courses for the instruction of students, and, on the other hand, medical men must show themselves more eager to benefit from the facilities for the study of cancer which already exist in the large centres of learning. If any doubt should still remain in your minds as to the extent to which delay in diagnosis influences the successful treatment of malignant disease, I commend you to the study of a most valuable and instructive series of reports by Dr. Lane Clayton[34] and others, issued under the auspices of the Ministry of Health during the last ten years.

Those who believe in the "unity" of the cancer problem naturally seek to find a solution for all diagnostic problems in a single test applicable to every type of malignant disease even at the earliest stage. If such a test were forthcoming its value would undoubtedly be great, and the discoverer would deserve to rank with Pasteur and Lister; but it is inherently most unlikely that a test of this kind could establish at once the site, size, histological structure, grade of virulence and radio-sensitivity of the growth, yet without a knowledge of all these the diagnosis must always be rated as imperfect.

Many attempts to provide such a universal diagnostic test for cancer have been made during recent years, and several of them have yielded a considerable percentage of accurate findings. Most of these tests rely on the assumption that the malignant cell adds certain abnormal substances to the blood-serum, or that these abnormal substances are produced by the body cells in response to the presence of the cancer cells.

Shaw-Mackenzie[35] was among the pioneers of such diagnostic tests. He maintained that in cancerous states the serum co-enzyme is decreased and, conversely, after removal of the growth it is increased, only to fall again in cases of recurrence. It is, however, only right to point out that the test has been severely criticized, especially when "positive." Normal healthy people, and those suffering from non-malignant diseases, often yield "positive" reactions to this test, and, in fact, whenever the fat-content of the serum is over 0·2 per cent. a positive reaction is found. When "negative" the test would appear to indicate that it is about four chances to one that cancer is absent, though Shaw-Mackenzie claimed a 95 per cent. accuracy for the test.

More recently modifications of the Abderhalden immunity reaction have been investigated with a view to establishing a reaction comparable to that of Wassermann. My colleague, the late Dr. H. J. B. Fry,[36] recorded in 1925 the results of such an investigation. He employed antigen from breast tumours, and his results accorded with the clinical and histological diagnosis in 75 per cent. of cases. Curiously enough, the diagnostic accuracy was higher for the more deeply-seated growths (such as those of the bowel) than for those of the skin. In sarcomata the index of accuracy was 86 per cent. As with Shaw-Mackenzie's test, a "positive" result was often obtained in other diseases, *e.g.* in febrile conditions and tuberculosis. Others have employed methods based upon the same principles, though modified in detail, but none of them has yielded a sufficiently high standard of accuracy to justify its employment as a diagnostic criterion warranting routine application.

There is another test which has been employed in various modifications by several observers based on the changes in the serum proteins which have been described as occurring in malignant disease. The Kahn,[37] Bendien,[38] Schmitz and Wulkow[39] tests are all dependent upon various methods of estimating the globulin-fraction in the blood-serum. It is fairly generally conceded that in about 80 per cent. of cases of cancer there is a demonstrable rise in the percentage of globulin in the blood-serum, but unfortunately an equally striking rise may occur in certain diseases in no way related to neoplasia, and it was this fact that was overlooked when Bendien's test was being checked a year or two ago. Great hopes were raised because a high degree of accuracy was reached in distinguishing the sera in cases of malignant disease from those of healthy controls, until it was realized that the test failed to differentiate between persons suffering from cancer and those the subject of certain serious (but not cancerous) diseases. Freeman[40] and his co-workers, and Smith, Holiday and Marrack[41] have demonstrated the futility of Bendien's method as originally described, not only in the respect of the flocculation factor, but also of the spectrographic examination of the precipitated proteids, but recently Cronin Lowe[42] has claimed a very high degree of accuracy for his own modification of Bendien's method.

In 600 unselected cases he calculates that the percentage error is only 4·25, and states that although pregnant women often give a positive reaction, he has good ground for regarding the method as reliable, not only as a qualitative test for malignant disease, but also quantitatively, as an indication of a rapidly-advancing case, or of a recurrence after treatment. This work has not been confirmed by any independent observer; on the contrary, such information as I have gleaned on the matter is unfavourable. Therefore, in view of the fundamental weakness of all such tests (since they are so frequently found positive in diverse diseases), I should be surprised if it survives the test of time and further experience.

Although I have explained my reasons for doubting the value of any general laboratory test for cancer, there are certain specific types of malignant disease at specified sites which can be detected with certainty by particular tests. Thus in carcinoma of the testis the Ascheim-Zondek reaction is obtainable from the urine (Hady),[43] and in fact becomes negative with the removal of the growth and reappears if it recurs. Ferguson,[44] of the Memorial Hospital, New York, has similarly established the diagnostic and prognostic value of the presence of intermedin—the hormone of the *pars intermedia* of the hypophysis—in the blood-serum, in cases of neuro-fibromatosis, neuro-sarcoma and melanotic sarcoma. In connexion also with the rare disease multiple myelomatosis the discovery of the specific (Bence-Jones) protein in the urine is diagnostic (McIntyre[45]).

Limited as may be the value of all these tests, are we justified in taking a pessimistic view of cancer diagnosis in general ? I think not. In the hands of those specially trained, diagnostic accuracy has been developed to the pitch that there are few cancers, however obscurely situated, which cannot be detected at any early stage, but in order to reach such high diagnostic standards the closest co-operation is needed between the various members of a team of experts working under the same roof. More—not less—centralization is required to ensure that facilities for the diagnosis of cancer, in the fullest sense of the term such as I have defined it earlier in this lecture, should be available. The cancer clinician must be accustomed to recognizing every type of pre-cancerous lesion, must be an expert in detecting by palpation early malignant masses, capable of employing with precision laryngoscope, bronchoscope, œsophogoscope, yes, to-day, even the flexible gastroscope of Schindler and Wolff,[46] to say nothing of the gastric camera, the thoracoscope, cystoscope, sigmoidoscope and vaginal speculum, and last, but not least, he should be trained in the recognition of all forms of malignant disease by naked-eye examination of the tumour in bulk. His associate, the radiologist, must be able to visualize with the fluorescent screen the early indications of abnormality in the alimentary canal which suggest malignancy, to interpret the subtle modification of the contours of the renal tract revealed by excretion-urography, to detect the peculiar type of calcification associated with certain tumours of the brain, to give a correct opinion of the numerous types of bone tumour in respect of their radio-sensitivity, and in innumerable other ways to confirm, modify or correct the clinician's findings. The pathologist who forms the third leg of this essential diagnostic tripod must not only be able to give a correct opinion on the histological nature of a tumour, but ought to be capable of grading its malignancy and judging of its probable response to radiotherapy. Such a diagnostic team is the product of years of concentration and continuity of effort, it cannot be created by the edict of bureaucratic bodies, however powerful, and it can never be duplicated to the degree which would permit of more than a strictly limited number of diagnostic centres being formed for the whole country. In the absence of a highly-organized system of cancer diagnosis such as I have outlined, far too much reliance tends to be placed on the examination of a portion of the tumour removed in an exploratory operation—the so-called biopsy.

The operation itself, unless conducted so that the whole tumour is removed with a reasonable margin of surrounding tissues, is liable to stimulate the growth or to cause its dissemination. The removal of a mere fragment of tissue may prove deceptive, the knife cut having failed to include any of the malignant cells. The portion excised may be so degenerate or atypical that an incorrect or doubtful opinion is arrived at. While, therefore, it is true that under favourable conditions the examination of biopsy material, both in the fresh state and after staining, may be occasionally helpful, we should rely more and more on a knowledge of gross pathology, and reserve biopsies for exceptional cases. When exploration is essential and the tumour deeply-seated, I prefer to use a fine hollow needle to obtain by aspiration the diagnostic material, rather than the usual open operation, and to give the pathologist all the time he needs for the examination rather than extract from him a hurried opinion. The soundest reason for a biopsy is that it enables the modern pathologist to grade the tumour according to the activity of its cells. This aspect of the diagnostic problem has not been as systematically pursued in this country as its merits deserve. I am satisfied that by using Broders'[47] grading method, which is based on the proportions of anaplastic and differentiated cells present, a more precise estimate of the potentialities of the tumour is obtained than by any mere qualitative description of the degree of malignancy of the tumour cells.

It ought not to be forgotten that the radio-therapeutist may be called on to aid us in cancer diagnosis as well as in treatment. The reaction of a tumour to one or more deep X-ray therapeutic doses may well help to resolve the doubt in certain difficult cases. Thus, in connexion with bone tumours, it will serve to differentiate between the cellular endosteal sarcoma (which regresses after exposure to X-rays) and chronic osteomyelitis (which is aggravated by similar doses). Summing up, one may say that though there is no justification for complacency, in recent years as great or greater progress has been made in the diagnosis of cancer as in any branch of cancer knowledge.

### TREATMENT.

*Surgery.*

Until early in the present century the scientific treatment of cancer was entirely governed by the development of surgical technique in response to the rapidly expanding knowledge of the pathology of neoplasms and their methods of dissemination. Thus, for cancer of the breast Stiles[48] and Handley[49] amplified and improved on the classical operation of Halstead, Crile[50] elaborated a method for extirpating secondary malignant cervical glands which has largely superseded that of Butlin, Miles[51] worked out a procedure for the resection of the malignant rectum which more fully fulfils the principles of surgical pathology than did Kraske's, Pólya[52] modified Billroth's form of gastrectomy and Wertheim[53] showed that it was possible to carry out a panhysterectomy in such a way as to conform to the surgical necessities of an infiltrating neoplasm of the cervix uteri, obtaining thereby many cures by purely surgical measures.

The notable successes of these surgical pioneers were not achieved without considerable mortality, to say nothing of the inevitable mutilation, but this has not deterred their followers from practising even more extensive operations in the attempt to realize the great surgical ideal in cancer therapy, viz. to remove the primary tumour and all of its extensions in one mass, without at any stage cutting through tissue containing malignant cells. Improvement in anæsthesia has done much to aid the surgeons in their endeavours, but even before the Great War it was becoming clear that the scope of surgical operations for cancer had well-nigh reached the limit which the human body could withstand.

Surgery, indeed, still has the premier place in the treatment of most forms of cancer, particularly those affecting the stomach, bowel, breast and brain, but it no longer is the sole therapeutic method available. Radium therapy, X-ray therapy singly or combined, or either or both in conjunction with surgical measures, must be recognized as having a place in cancer treatment, and at certain sites, *e.g.* the cervix uteri, to have largely superseded purely surgical procedures.

*Irradiation.*

The revival of radium therapy, after its original eclipse, started little more than ten years ago, and in this country really systematic work on a large scale scarcely covers half that period, so that we are dependent to a great extent on the reports from Paris, Stockholm, and New York for most of the statistical information relating to the numbers of five to ten year survivals. Enough has been learnt, however, to cause us to emphasize the difficulties and dangers of radium therapy rather than the reverse, and to raise doubts as to the wisdom of endeavouring to popularize this form of treatment by multiplying the number of hospitals and clinics where it can be applied. It is useless, or positively dangerous, for generous donors to supply institutions with large amounts of radium unless a trained staff of physicists, radium officers and technicians, pathologists and surgeons is available, to say nothing of the special buildings and plant which cannot be easily improvised.

The Radium Commission has so far wisely avoided this error by limiting the number of approved "National" centres to large towns with Universities or Medical Schools, but there are, unfortunately, many independent institutions where radium therapy is essayed without the necessary minimum personnel, and therefore unsatisfactory results are bound in the end to arise, and thus unjustly discredit this type of treatment.

Radio-sensitivity is now known to be the real basis of successful radium treatment. It is, however, still to some extent a matter of discussion as to what part is played in radio-sensitivity by the cancer cells themselves, by the reticulo-endothelial cells, and by the blood-vessels in and around the tumour cells respectively; but there is strong evidence that the macrophage is a very prominent factor in the production of favourable responses to gamma rays. It is also believed that the gamma rays are more effective in tumours the cells of which are actively dividing, or about to divide, and in those with a soft, vascular, delicate stroma.

Regaud and Lacassagne[54] have worked out the radio-sensitivity of a large number of normal tissues and of the cells of some neoplasms, but although this work has been amplified by others recently, we can still only speak in very general terms of the radio-sensitivity of most neoplasms. Thus Haagensen[55] found in opposition to the law of Bergonié and Tribondeau, that in adenocarcinoma of the thyroid the most rapidly growing and anaplastic types were uniformly radio-resistant.

Closely related to the subject of radio-sensitivity is that of radium dosage, and this has been one of the greatest stumbling-blocks in radium therapy. The standards of dosage have, until recently, been milligram-hours or millicuries destroyed—neither of these methods has any real scientific or comparable value, but happily we are now rapidly approaching the stage when it will be possible to estimate dosage in standard *r*-units as now employed in deep X-ray therapy. Mayneord,[56] for example, has calculated that 1 mg.-hr. at one centimetre distance from a point source is approximately 9 *r*, and this figure has been confirmed in several ways by other observers. It has been customary in many centres to express the dose of gamma or X-rays in terms of the skin erythema dose (S.E.D.), but since this may be 50 per cent. more in the case of the scalp than elsewhere, and greater in skin poorly supplied with blood than it is in well-nourished skin areas,[57] it is obvious how urgent it is to provide a universal standard for gamma radiation, as has been done recently for X-radiation. I can only briefly refer to a few points in radium therapy which appear to me to represent important advances.

In employing the *interstitial method*, by " needling " or by " radon " seeds, there has been a tendency in certain clinics to increase the screenage from the more usual 0·5 mm. Platinum to 0·8 mm., and improved results are claimed in the avoidance of damage to cartilage and bone, the capacity to give more prolonged doses and in the actual benefits which follow.

With *surface applications* the main advance in recent years has been in the more accurate distribution of the radium so as to give a uniform dosage. The necessary calculations for this in connexion with plane surfaces have been carried out by Mayneord[58] and he is extending the work to surfaces of a more complicated form.

*Beam Therapy.*—On the Continent and in the Memorial Hospital, New York, " bombs " of four grammes or more have been in use for several years. In this country there has been little opportunity to use so large an amount. A 5-gramme unit is, I am glad to say, to be set up immediately under the control of the Radium Beam Research Committee, and important developments are to be expected at the end of the two years' experimental period.

We have been using a 1-gramme unit successfully for some years at the Cancer Hospital, and others of this size are now being worked with at University College Hospital and at the Middlesex Hospital, while a 2-gramme bomb is employed at the Westminster Hospital. The great virtue of the " bomb " is that by adopting several " ports " of entry it is possible to deliver a depth-dose of uniform intensity to growths which are almost or quite inaccessible to other methods of radium therapy, and which are hardly within the realms of practical surgery.

I can only lightly touch on the triumphs of the gamma-ray treatment in cancer and the indications for its use. It is the best method of treating (with a view to cure) epithelioma of the skin, lip, mouth, tongue, pharynx and larynx, the cervix uteri, the vulva and penis, anus, some cancers of the breast and rectum and certain bone sarcomata.

The " beam " can be employed to cure or to palliate growths of the hypopharynx, pyriform fossa, tonsil and pharyngeal wall which formerly were quite hopeless or amenable only to operative measures which were dangerous, mutilating and frequently ineffective. It must not be forgotten, however, that even by this new method violent reactions may ensue, and that the patient may suffer extreme discomfort and dryness of the mouth and dysphagia. Therefore it should not be employed unless the growth is of a reasonable size, or alternatively, in the more advanced cases, the dosage should be reduced.

Speaking generally, effective as gamma-ray treatment may be for primary growths, it is seldom possible to prevent glandular deposits by the methods at present in vogue. It is, I think, probable that the results of massive radiation from a 5-gramme bomb, when this information eventually is available, may cause us to change our outlook on this matter. In the meantime, the trend of opinion is in favour of a systematic block-dissection of glandular drainage areas whenever these are accessible and the condition of the patient justifies it. My own experience so far is emphatically that glandular metastases are the province of the operating surgeon, and that the wider and more systematic the extirpation of these glands the better the eventual result.

While we must rate radium therapy very high in the treatment of certain cancers, as I have already admitted, the difficulties, dangers and inconveniences of the method are now only too well known. If it were certain that the radiation from radium is alone of real value in cancer therapy, there would be little justification for seeking substitutes for it, but there is abundant evidence that radiation of longer wavelengths than the gamma-rays is of considerable efficacy in destroying cancer cells; in fact, the optimum wavelength for every type of growth is not known. It is, however, believed, and with some justification, that the shorter waves are of greater efficacy than the longer, and that the nearer they approach in quality the gamma-rays from radium the more valuable therapeutically.

For this reason attempts have been made recently to develop X-ray therapy, utilizing higher and higher voltages so that harder and harder rays are produced. Assuming a biologically similar effect, the advantages of deep X-ray therapy over radium therapy are that an apparatus costing about £3,000 can be made to do the work of 170 grammes costing about £3,000,000 without any extra expense in the way of running costs. At the Cancer Hospital we have been working with a voltage approximating to 400 k.v. At the Memorial Hospital, New York, 700 k.v. have been used for about three years, and there seems to be no reason why eventually the voltage should not be raised sufficiently to bridge almost entirely the gap which still exists between gamma-rays and the hardest X-rays yet utilized in cancer treatment. Already the results obtained by the higher voltages have shown a great advance on those gained by the usual 180—220 k.v. apparatus, not only when used as a substitute for gamma radiation, but also when they are employed in conjunction. I can foresee the time when deep X-ray therapy will to a large extent, if not wholly, replace radium therapy, but I am not sufficient of a prophet to foretell how soon this will come about. At the Memorial Hospital, New York, careful comparisons have been made between the physical, biological and therapeutic effects of the X-rays of 200 k.v., 700 k.v. and gamma-rays from radium (G. Failla McNalton et alii, 1933).[59] It is impossible to summarize their results satisfactorily, but in respect to cancer of larynx, pharynx, hypopharynx and tonsil (which are accessible and easily observed sites), working with divided doses and 700 k.v., it was found that the local and general discomfort were both reduced as compared with what happens with 200 k.v. and with gamma-rays from radium. It is not possible at present to say whether this conclusion can be expanded into a generalization applicable to other types of growths at various sites. Much in this connexion is to be expected from the adoption of the universal *r*-unit for X-ray dosage, as this will enable much more reliable comparisons to be made between these therapeutic measures.

It has been shown that deep X-ray therapy, combined with gamma-ray therapy, is capable of giving a higher percentage of cures or a more complete palliation in the treatment of cancer at certain sites, *e.g.* carcinoma of cervix, than is gamma-ray therapy alone. As an adjunct to surgical measures there can be no doubt whatever from my own experience and that of others that deep X-ray therapy, especially from high voltage apparatus, is essential if the best results are to be arrived at. I have some evidence that in acute cancer of the breast in young or lactating women deep X-ray therapy will render what appeared an inoperable case operable, while after operation it can be applied far more effectively against recurrence than any form of gamma radiation, except, perhaps, the " beam " from a 4- or 5-gramme unit. Dawson and Tod[60] (1933) recently collected some reliable figures in relation to carcinoma of the breast which show that the combination of operative methods with post-operative X-ray therapy increases the five-year survival rate by nearly 10 per cent. Comparable figures could be quoted, I believe, in relation to cancer of the tongue, lips, floor of mouth, palate, pharynx, etc., in all of which, even after the disappearance of the primary lesion following gamma radiation and the most radical block dissection of the cervical glands, deep X-ray therapy is needed to destroy outlying cancer cells in the remoter lymphatics, and especially in the zone of tissue between the original growth and the area embraced by the block dissection.

*Chemotherapy.*

Ehrlich's successful attack on the Treponema has stimulated the search for some compound which should destroy the cancer cell and prove as effective as does salvarsan in the control of syphilis. Unfortunately, the theoretical basis for such a research is less sound than was Ehrlich's, since there is so little evidence of any physico-chemical differences between the normal and the cancer cell, though it is true that Warburg[61] has described powerful glycolytic powers for some cancer cells. This property is not, however, specific to cancer cells, being exhibited also by the retinal cells and by those of the placenta.

It is possible that the various chemical substances which have been tried in cancer therapy act—

(1) Directly, *i.e.* on the cancer cell itself; or

(2) Indirectly, by increasing the power of the body cells to resist the growth of the cancer cell.

Caspari,[62] for example, conceived that the metallic salts he employed became precipitated in the tumour and increased autolysis, with the formation of necro-hormones, which, carried into the blood-stream, stimulated the reticulo-endothelial cell system. Wassermann believed that tumour cells have an affinity for certain chemical compounds (*e.g.* eosin), and that this could be utilized to carry selenium to the tumour. Ludford's[63] experiments on dyes suggest that they are deposited, not in actively growing cancer cells, but in those which are degenerating, and that the bulk of the deposit is in the connective tissue in and around the tumour masses—especially the primary. The Golgi apparatus appears to play an important part in the segregation and elimination of the dyes.

Successful chemotherapy demands (1) an intravenous administration, in order that all secondary deposits may be affected as well as the primary lesion ; (2) the agent should be deposited chiefly in the tumour cells and the connective tissue in the neighbourhood, a condition which Kawata's experiments render very unlikely, since in cancerous mice treated with lead the latter is found mainly in the liver and very little in or near the cancer cells ; (3) the ratio of the toxicity of the compound for body and for tumour cells respectively should not be less than 1 : 3—an almost insuperable difficulty.

With the object of finding such an ideal form of chemotherapy numerous metallic and other compounds have been tested. With Wassermann's original selenium injection the survival rate was only 3—5 per cent. owing to the toxicity of the drug.

Neuberg and Caspari[64] employed copper, selenium, tin, zinc, cobalt and silver sometimes in colloid form, but while necrobiotic changes were observed in the tumours, the mortality was prohibitive. Curiously enough, in view of Todd's work, they noted a stimulating effect in the tumours with small doses of the various colloidal metals. Keysser systematically re-investigated the action of selenium in 1914 in animals, and could find no evidence that it has any action if the tumour is small, though it may produce shrinkage in already degenerating neoplasms.

Clinically Touche in 1913,[65] reported good results with selenium therapy, more especially as a palliative method.

Blair Bell[66] in 1922 observed benefit from the use of approximately 0·5 gramme to 0·8 gramme of lead in a concentration of 0·5 per cent. The preparation he employed was alleged to be a colloidal one, obtained by the Bredig process. The method of administration was modified from time to time after it was first introduced, chiefly by spreading the injections over a more extended period than at first.

Some cures were claimed, in addition to a considerable percentage of cases of arrest of the disease, and many in which the pain had been alleviated. In some of the cases radiotherapy and surgical extirpation were employed in addition to lead therapy. Many independent observers, including Hume,[67] Wyard,[68] Burton Simpson[69] and Thomson,[70] have failed to obtain such favourable results, and the majority of these authors report a high morbidity and prohibitive mortality. I can myself testify that at the Cancer Hospital lead treatment was given a most careful and exhaustive trial, and that the results were deplorable. Todd's colloidal lead selenide is much less toxic than Blair Bell's preparation, and appears to be as successful. It is very interesting, and perhaps significant, to note that lead therapy has apparently been entirely abandoned in Liverpool, where it was initiated and actively persisted in for so long a period.

Copeman, Coke and Gouldesbrough[71] have sought to enhance the effect of X-rays by adding a fluorescing substance which they assume produces a secondary radiation in the neighbourhood of the tumour cells, thus adding to the lethal effect of the primary radiation. They inject 20 cc. of 5 per cent. Sodium Fluorescine intravenously or paint it on to the surface of superficial tumours.

Dr. Todd's[72] recent work in cancer-therapy constitutes one of the most earnest and thoroughly systematic attempts to treat inoperable or otherwise apparently incurable growths. The following is an outline of the method as practised at present, though perhaps it is supererogatory for me to describe it to a Bristol audience.

Dr. Todd believes that such beneficial effects as X-rays and gamma-rays may have in the treatment of malignant disease are the result of their action on the connective tissue in and about the neoplasm itself, this tissue being regarded as inhibitory to neoplastic growth and termed "junction" tissue. His experience in human beings and his experiments in mice lead him to the conclusion that X-rays and gamma-rays applied in the usual dosage on subjects impregnated with selenium colloids may actually stimulate the growth of the cancer, and he explains this as due to a destructive or paralytic action on the "junction" tissue.

Acting on this hypothesis, Todd has abandoned the use of lead selenide (D4S) in conjunction with massive radiation, and has substituted for it a sulphur-selenium colloid containing no metal, combining this with X-radiation. The colloid is administered intravenously once weekly, or fortnightly in specially sensitive cases, and forty-eight hours later the dose of X-rays is applied. Todd concludes that the particles of selenium are acted upon in the "junction" tissue by the penetrative radiation, the effect being termed ionization, and the result held to be a stimulation of the junction tissue to check neoplastic growth.

Experiments are being carried out in his laboratories to show whether short wireless waves, approximately of two metres wave-length, will induce this ionization effect, and for some time he has employed a selenium colloid therapeutically which is radio-active in order to induce a state of permanent ionization in the "junction" tissue.

Dr. Todd claims :—

1. About 50 per cent. "3-year apparent cures" of inoperable cancer of the breast and of the ovary.

2. About 60 per cent. "3-year apparent cures" in sarcoma generally.

Speaking as one who has had the opportunity of studying malignant disease in its inoperable forms from beginning to end in the chronic wards of the Cancer Hospital, I am obliged to offer certain criticisms of Todd's views. It is by no means rare for cancerous masses, both primary and secondary, to diminish in size, ulcerating growths may heal up wholly or partially, and patients may improve in health and increase in weight even when no specific attempt is made to treat the neoplasm. So far as I am concerned I have never known a single case of idiopathic regression or apparent arrest of tumour growth in which eventually recurrence did not manifest itself, but sometimes the quiescent interval is prolonged.

It is only fair to state, too, that there are records —some highly circumstantial—of nearly 400 cases of complete spontaneous cure of cancer.

I can, however, testify to the optimistic outlook of the patients under Dr. Todd's care and to the subjective improvement which many of them report.

My main ground for criticism is that there is no evidence that effective ionization (or secondary radiation) occurs with the doses of selenium which are employed. Mayneord and Burrows'[73] work indicates that it would require a dose of the selenium colloid several hundred times as great as that administered by Todd to produce the minimal effective ionization, even allowing for a high selective concentration of the colloid in and around the tumour cells and stroma. Finally, the method of checking the progress of treatment by Rona's serum-lipase estimation has been clearly demonstrated to be quite fallacious.

*Serum Therapy.*

The last therapeutic method to which I need refer is that of Lumsden. Here we are dealing with an attempt to provide a universal cure for cancer—chimerical, if we accept the main thesis of my lecture—but one extremely attractive to many cancer-therapeuticians and to all laymen.

Lumsden[74] is endeavouring to prepare a serum which will stimulate the natural powers of resistance of the normal tissues to the spread of cancer cells. He claims that antibodies of this nature occur in the blood-serum, and that the reticulo-endothelial cells produce a cytase which damages the malignant cells and they then become the prey of the antibodies. He has obtained sera which will destroy cancer cells in animals, but the amount of antibody is so small that unless it can be concentrated it is ineffective in man. In animals the concentration of the antibody is effected by shutting off the circulation to the tumour temporarily. It is claimed by Lumsden that by fractioning off the euglobulin of the serum the concentration of the antibodies which adhere to the euglobulin is thereby increased tenfold. This concentrated serum is now being employed in treating inoperable cases of cancer at the London Hospital, but the introducer of the method admits that much research is still necessary, and that many years may elapse before the method is perfected.

Mr. Vice-Chancellor, in concluding this lecture, and in thanking my hearers for their courteous attention, may I be allowed to stray beyond the limits imposed by my own choice of title? Only a few days ago I returned from an International Conference on Cancer, and there I could not help being exalted by the optimism of the younger school, both research workers and clinicians, and depressed by the slightly cynical pessimism of the older school. I believe that the young men are right, that with the aid of vision and courage the causes of cancer will be discovered, and that such knowledge will inevitably lead to the formulation of methods, not so much of cure, as of prevention. The essential clues to the cancer mystery lie in the biochemistry of the cell, and in the amazing powers of the hormones of the various endocrine glands, which are only now beginning to be realized, but I am confident that with the broadening of our knowledge of these subjects will come the clarification of the whole cancer problem.

### REFERENCES

1 Farmer, J. B., Moore, J. E. S., and Walker, C. E., *Brit. Med. Jour.*, 1903, ii. 1,664

2 Handley, W. S., *The Genesis of Cancer;* London: Kegan Paul & Co. Ltd., 1931.

3 Gye, W. E., and Purdy, W. J., *The Cause of Cancer;* London: Cassell & Co., 1931.

4 Fibiger, J., *Ztscht. f. Krebsforsch*, 1913, xiii. 217.

5 Ross, J. M., *Jour. Path. and Bact.*, 1932, xxxv. 899.

6 Rehn, L., *Arch. f. Klin. Chir.*, 1895, l. 588.

7 Yamagiwa, K., and Ichikawa, K., *Jour. Cancer Research*, 1918, iii. 1.

8 Daels, F., *Bull. de l'Assoc. franç. pour l'Etude du Cancer.*

9 Jonkhoff, A. R., *Zeits. f. Krebsforsch*, 1928, xxvi. 32.

10 Findlay, G. M., *Lancet*, 1928, ii. 1,070.

11 Heyninx, A., *Bruxelles. Méd.*, 1928, viii. 1,542.

12 Steinbrück, C., *Berl. tierärtzl. Woch.*, 1929, xlv. 525.

13 Berenblum, I., *Brit. Jour. Exper. Pathol.*, 1929, x. 179.

14 Twort, C. C., and Twort, J. M., *Jour. Indust. Hyg.*, 1931, xiii. 204

15 Leitch, A., *Brit. Med. Jour.*, 1922, ii. 1,104.

16 Maisin, J., and François, A., *Ann. de Méd.*, 1928, xxiv. 455.

17 Watson, A. F., *Cancer Review*, 1932, vii. 445.

18 Kennaway, E. L., personal communications, and see also references 9 and 13; and Cook, J. W., Hieger, I., Kennaway, E. L., and Mayneord, W. V., *Proc. Roy. Soc. Brit.*, 1932, cvi. 455.

19 Hieger, I., *Biochem. Jour.*, 1930, xxiv. 505.

20 Cook, J. W., *Proc. Roy. Soc. Brit.*, 1932, cxi. 485.

21 Cook, J. W., Hewitt, C., and Hieger, I., *Nature*, 1932, cxxx. 926.

22 Ibid., *Jour. Chem. Soc.*, 1933, April, p. 395.

23 Mayneord, W. V., personal communications.

24 Wells, H. G., *Amer. Jour. Cancer*, 1931, xv. 1919.

25 Slye, M., *Jour. Cancer Research*, 1928, xii. 83.

26 Tyzzer, E. E., *Jour. Med. Research*, 1907, xvii. 199.

27 Little, C. C., *Jour. Cancer Research*, 1928, vii. 30.

[28] Haldane, J. B. S., *Nature*, 1933, cxxxii. 265–267.
[29] MacCarty, W. C., *Amer. Jour. Cancer*, 1933, xvii. 25–33.
[30] De Vries, W. M., *Cancer Control*, The Surgical Publishing Co., Chicago, 1927, p. 217.
[31] *Reports*, Imperial Cancer Research, 1905, ii. part 1.
[32] Bilz, G., *Zeitsch. f. Krebsforsch*, 1922–23, xix. 282.
[33] Reiman, S. P., and Safford, F. H., *Amer. Jour. Cancer*, 1931, xv. 1,338.
[34] Lane-Clayton, J. E., *Reports*, Ministry of Health, London, No. 28.
[35] Shaw-Mackenzie, J. A., *Jour. Physiol.*, 1911, xlii. xi.–xvi. ; *Proc. Roy. Soc. Med.*, 1912, v., Therap. Sec., p. 152.
[36] Fry, H. J. B., *Brit. Med. Jour.*, 1925, ii. 4.
[37] Kahn, H., *Klin. Woch.*, 1924, iii. 920 ; *ibid.*, 1925, iv. 178, 222.
[38] Bendien, S. G. T., *Spezifische Veränderungen des Blutserums*, Jena : Gustav Fischer. 1931.
[39] Schmitz, A., and Wulkow, F., *Biochem. Ztschr.*, 1932, ccxlv. 408.
[40] Freeman et al, *Med. Jour. Australia*, 1931, ii. 778.
[41] Smith, F. C., Holiday, E. R., and Marrack, J., *Lancet*, 1931, ii. 507.
[42] Lowe, E. C., *Brit. Med. Jour.*, 1933, i. 407 ; *Brit. Jour. Radiol.*, 1933, vi. 207.
[43] Hady, *Zentralbl. f. Gynäk.*, 1931, lv. 912.
[44] Ferguson, personal communication.
[45] McIntyre, W., *Trans. Med.-Chir. Soc.*, 1850, xxxiii. 211.
[46] Schindler, R., *Münch. Med. Woch.*, 1932, lxxix. ii., 1,268.
[47] Broders, A. C., *Med. Jour. and Record*, 1925, cxxi. 133.
[48] Stiles, H. J., *Brit. Med. Jour.*, 1899, i. 1,452.
[49] Handley, W. S., *Cancer of the Breast*, London, 1906.
[50] Crile, G. W., *Surgery*, W. W. Keen, London, 1908, vol. iii. 328–333.
[51] Miles, W. E., *Brit. Jour. Surg.*, 1914–15, ii. 292.
[52] Pólya, E., *Zeutralbl. f. Chir.*, 1911, 892.
[53] Wertheim, E., *Arch. f. Gynäk.*, 1900, lxi., 627.
[54] Regaud, C., and Lacassagne, A., *Radiophysiologie*, 1927–29, i. 1.
[55] Haagensen, D., *Amer. Jour. Cancer*, 1931, xv. 2,063.
[56] Mayneord, W. V., and Roberts, J. E., *Brit. Jour. Radiol.*, 1933, vi., 321 ; *Med. Research Council Special Report No.* 186, London, 1933.
[57] Mottram, J. C., *Brit. Jour. Radiol.*, 1932, v. 643.
[58] Mayneord, W. V., *Acta Radiol.*, 1933, xiv. 95.
[59] Failla, G. McNalton et alii., *Amer. Jour. Cancer*, vol. xxix., 1933, No. 3, 293.
[60] Dawson, E. K., and Tod, M. C., *Edin. Med. Jour.*, 1933, xl. 157.
[61] Warburg, O., *The Metabolism of Tumours*, London, 1930.
[62] Caspari, W., *Report International Conference on Cancer*, London, 1928, p. 199.
[63] Ludford, R. J., *Proc. Roy. Soc. Brit.*, 1929, civ. 493.
[64] Neuberg, C., and Caspari, W., *Deutsch. med. Woch.*, 1912, xxxviii. 375.
[65] Touche, M., *Bull. et mém. Soc. méd. d'hôp. de Paris*, 1913, xxxv. 451.
[66] Bell, W. Blair, *Lancet*, 1922, ii. 1,005.
[67] Hume, J. B., *Report International Conference on Cancer*, London, 1928, p. 239.
[68] Wyard, S., *Brit. Med. Jour.*, 1928, i. 838.
[69] Simpson, B. T., *Report International Conference on Cancer*, London, 1928, pp. 221, 244.

[70] Thomson, A. P., *Birmingham Med. Review*, 1928, iii. 29 ; iv. 141.
[71] Copeman, S. M., Coke, F., and Gouldesbrough, C., *Brit. Med. Jour.*, 1929, ii. 233.
[72] Todd, A. T., *Lancet*, 1930, ii. 389 ; *Chemotherapeutic Res. on Cancer;* Bristol : J. W. Arrowsmith Ltd. 1928.
[73] Mayneord, W. V., and Burrows, H., *Brit. Jour. Radiol.*, 1931, iv. 369, 454.
[74] Lumsden, T., *Tenth Ann. Report B.E.C.C.*, 1933, p. 222.

## Figures and Tables

**Figure f1:**
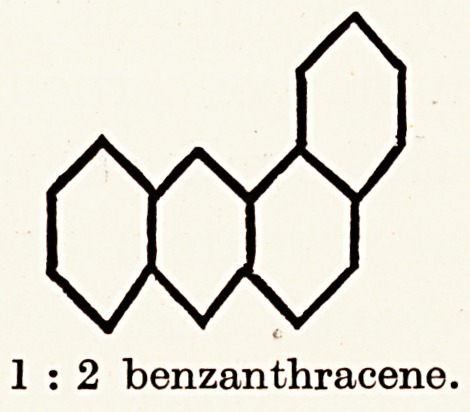


**Figure f2:**
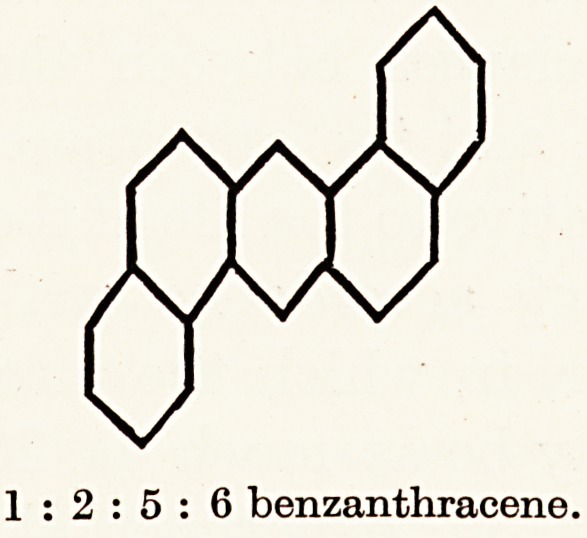


**Figure f3:**